# Type IV‐A choledochal cyst with choledocholithiasis in an adult female: A case report

**DOI:** 10.1002/ccr3.7992

**Published:** 2023-09-27

**Authors:** Pratik Bhattarai, Bishowdeep Timilsina, Prasun Khanal

**Affiliations:** ^1^ Manipal College of Medical Sciences Pokhara Nepal; ^2^ Oxford University Clinical Research Unit Nepal Nepal; ^3^ Department of General Surgery Manipal Teaching Hospital Pokhara Nepal

**Keywords:** Choledochal cyst, Choledocholithiasis, hepaticojejunostomy, Todani classification

## Abstract

A choledochal cyst is a rare congenital anomaly of the biliary system, characterized by bile duct cystic dilatation, typically affecting the common bile duct. Choledochal cysts are generally categorized using the Todani classification system. The typical symptoms are jaundice, abdominal masses, and recurrent abdominal pain. As most cases are diagnosed in children, adult presentations are uncommon and often associated with complications. A 22‐year‐old female patient complained of severe abdominal pain and vomiting for 5 days, with signs of jaundice. Her abdominal ultrasound revealed fusiform dilation of the extrahepatic common bile duct with multiple calculi in its distal‐most part. On CT cholangiogram of the abdomen, Type IV‐A Choledochal cyst with non‐obstructive choledocholithiasis was found. Although rare, choledochal cysts are a well‐known clinical entity. It is essential to diagnose and treat patients because they may develop complications. Cholecystectomy combined with Roux‐en‐Y hepaticojejunostomy is the preferred treatment for Type IV‐A choledochal cysts. Since choledochal cysts in adults are uncommon, early detection and treatment are essential to avoid serious complications. Ultrasonography (USG), Magnetic resonance cholangiopancreatography (MRCP), and Computed tomography (CT) can provide a diagnosis.

## Graphical abstract

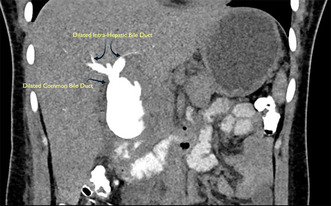

## 1 INTRODUCTION

Congenital bile duct malformations known as choledochal cysts are abnormal, disproportionate cystic dilatations of the biliary channel.^1^ The detection of cases in childhood accounts for around 80%; thus, adult presentations are uncommon and often come with complications like cholangitis, stone development, cyst rupture, secondary biliary cirrhosis, obstructive jaundice, and malignancy (cholangiocarcinoma).^2^ The Todani Classification divides choledochal cysts into Type I–Type V.^1^ Multiple cystic dilatations of the intra and extrahepatic biliary channels characterize type IVA choledochal cysts, the second most common type after Type I.^1^ The classic triad includes pain, jaundice, and an abdominal mass; however, less than 20% of patients will present with all three findings.^3^

Medical imaging has evolved and improved, making diagnosing more cases possible. Abdominal ultrasonography (USG), computed tomography (CT), magnetic resonance cholangiopancreatography (MRCP), endoscopic retrograde cholangiopancreatography (ERCP), and endoscopic ultrasound are the most often requested investigations.^2^ The treatment consists of a total cyst resection and Roux‐en‐Y hepaticojejunostomy.^2^ This case report underlies a Type IV‐A choledochal cyst in a female complicated with choledocholithiasis.

## 2 CLINICAL PRESENTATION

A 22‐year‐old female from Nepal presented with a chief complaint of abdominal pain for 5 days. Her pain was in the epigastric area, colicky in nature, and was continuous. The pain was associated with multiple episodes of vomiting, containing food particles and yellow in color. However, she denied fever, bowel changes, and weight loss. On physical examination, the patient had icterus, but no pallor, and her vitals were within normal limits. Abdominal examination revealed mild epigastric tenderness, with no rebound tenderness or Murphy's sign. All other system examinations were within normal limits. Her previous medical history was insignificant, with no comorbidities. She had no surgeries in the past. She had not taken any medicine before except an analgesic for the pain. Her family history was not significant. Laboratory investigations were sent, which showed hemoglobin of 10.31gm/dL with low mean corpuscular volume (MCV), mean corpuscular hemoglobin (MCH), and mean corpuscular hemoglobin concentration (MCHC); Serum bilirubin 2.8 mg/dL with dominant conjugated bilirubin. The rest of the laboratory data are shown in Table 1.

**TABLE 1:** Laboratory results.

Test	Results	Reference range	Units
WBC count	10.31	4–11	*10^3^/cu mm
Neutrophils	84	40–80	%
Lymphocytes	10	20–40	%
Hemoglobin	11.6	13–17	gm/dL
MCV	73.1	83–100	fL
MCH	24.9	27–32	pg
MCHC	34.1	40–50	%
Platelets	264	150–400	10^3^/cu mm
Total bilirubin	2.8	0.2–1.3	mg/dL
Conjugated bilirubin	2.0	0–0.3	mg/dL
Unconjugated bilirubin	0.8	0–1.1	mg/dL
AST	58	14–36	U/L
ALT	88	0–35	U/L
ALP	233	38–126	U/L
Total protein	4.6	6.3–8.2	gm/dL
Albumin	2.2	3.5–5.0	gm/dL
Globulin	2.4	2.0–3.5	gm/dL
Serum lipase	115	30–110	U/L
Serum amylase	62.1	70–140	mg/dL
HCV	Negative		
HBsAg	Negative		
HIV	Negative		
PT/INR	15.3/1.3		Seconds

Abbreviations: ALP, alkaline phosphatase; ALT, alanine transaminase; AST, aspartate aminotransferase; HCV, hepatitis C virus; HIV, human immunodeficiency virus; MCH, mean corpuscular hemoglobin; MCHC, mean corpuscular hemoglobin concentration; MCV, mean corpuscular volume; PT/INR, prothrombin time/international normalized ratio; WBC, white blood cell.

**FIGURE 1:**
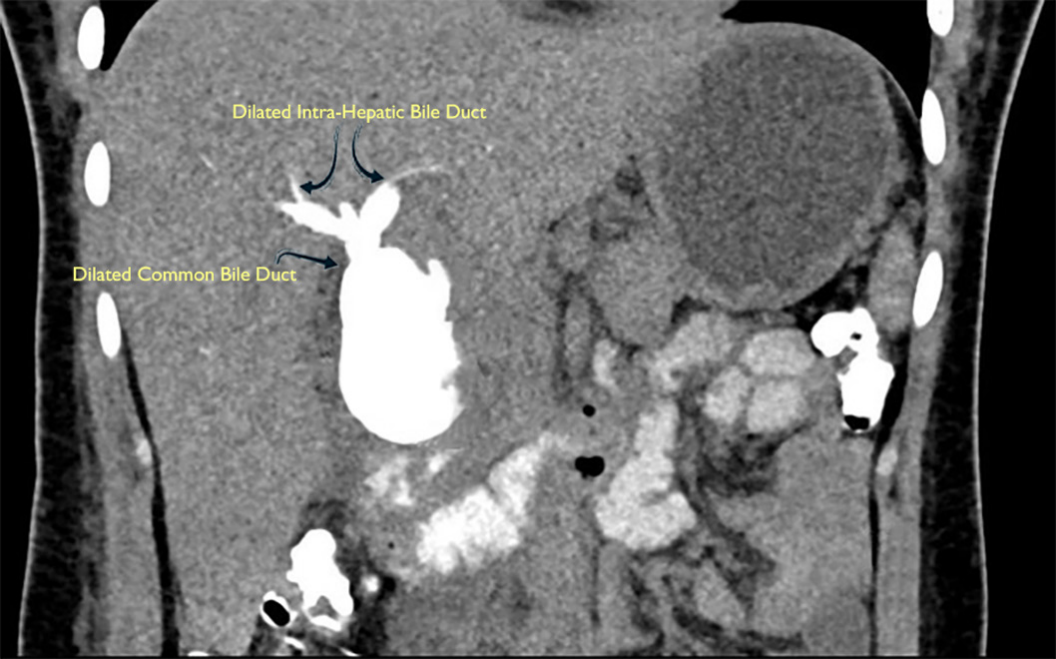
CT cholangiogram showing fusiform dilatation of the entire common bile duct (single arrow), with intrahepatic bile duct dilatation (two arrows).

Abdominal USG showed intra and extrahepatic common bile duct (CBD) dilation with multiple calculi in the distal‐most part with a normal gallbladder. The CT cholangiogram showed fusiform dilatation of the CBD, measuring approx. 5.3 × 3.3 cm in size with an extension of dilatation to the intrahepatic bile duct (Figure 1). Also, multiple tiny microcalcifications in the dependent portion of distal CBD were seen. The pancreatic duct was normal, and no anomalous pancreaticobiliary junction was detected. Most importantly, there were no distinct indications of malignancy.

Thus, the final diagnosis was made with Type IV‐A choledochal cyst with non‐obstructive choledocholithiasis. Fluid resuscitation with normal saline was given initially. Other treatments included proton pump inhibitors, antiemetics, and analgesics. Following treatment, the patient showed clinical improvement.

**FIGURE 2:**
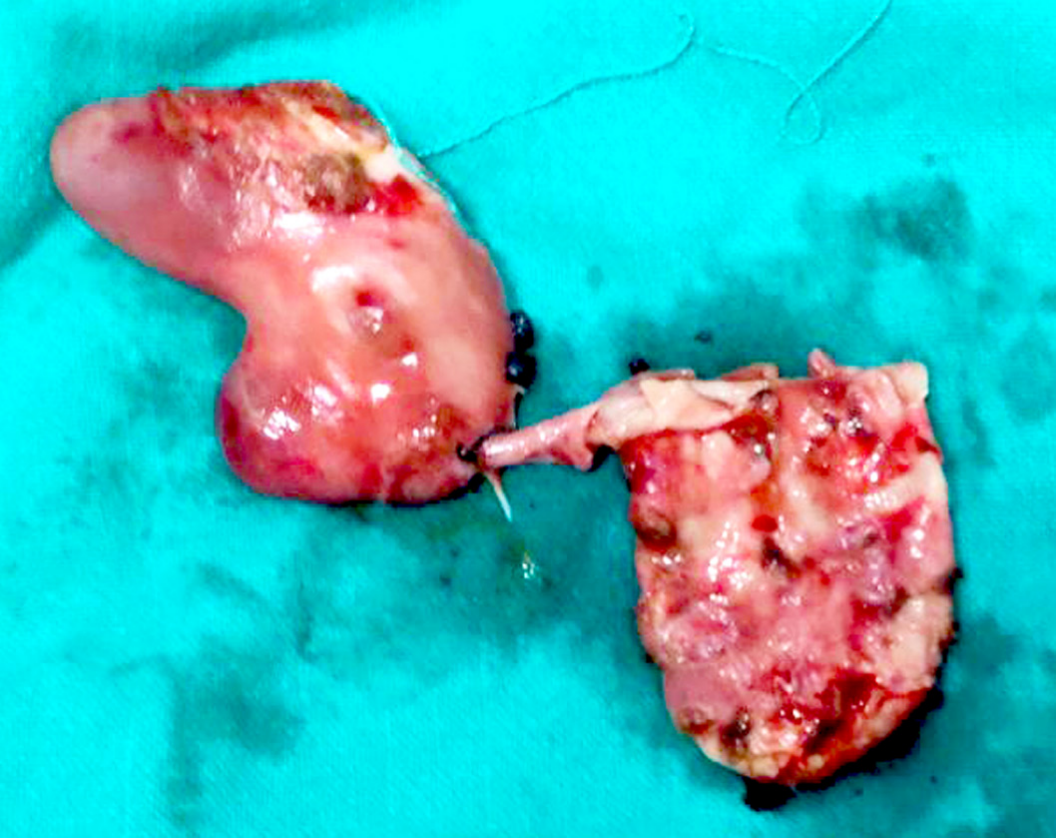
Resected common bile duct cyst and gallbladder.

Two days later, the patient underwent surgery performed by a general surgeon. The procedure began with the surgeon making a Kocher's incision. There was a choledochal cyst measuring approximately 5 × 3 cm along its entire course, tapering at the infra‐duodenal portion. The choledochal cyst was transected proximally at the level of the hilum, where there was an abrupt change in caliber, and distally in the cyst's intrapancreatic waist, without injuring the pancreatic duct. The cyst was then wholly excised, and cholecystectomy (Figure 2) with reconstruction by Roux‐en‐Y hepaticojejunostomy was done. Multiple calculi were found inside the choledochal cyst. The liver, bowel, and pancreas were found to be healthy.

The excised choledochal cyst was sent for histopathological examination, which showed evidence of inflammation and hyperplasia, a lack of biliary mucosa in the cyst. Furthermore, there was no evidence of premalignant lesion, confirming no malignancy.

The postoperative period was uneventful, being discharged on the eleventh day after surgery. The patient was followed up for a year at an outpatient clinic with an uneventful recovery with no malignancy or stricture.

## 3 DISCUSSION

A choledochal cyst is a rare congenital biliary system abnormality involving any bile duct segment. It presents as cystic dilatation of bile ducts, usually occurring in the common bile duct.^4^ According to a Finland report, bile duct cyst prevalence has grown over the past 40 years from 1:128000 to 1:38000. While most instances have historically been reported in children, some investigations have shown similar results in adults and children.^2^ Numerous theories have been put forth on the origin of the choledochal cyst. However, only some of these theories explain the various choledochal cyst types. The first and most widely recognized explanation is related to the abnormal union of the pancreaticobiliary duct.^4^ Choledochal cysts are generally classified into five subtypes by the Todani classification. Type I bile duct cyst consists of Type I A (cystic dilatation of the CBD), Type I B (saccular dilatation of the CBD), and Type I C (fusiform dilatation extending to the common hepatic duct). Type II bile duct cysts are extrahepatic duct diverticula. Type III is cystic dilatation of the intraduodenal portion of common bile duct (choledochocele). Type IV is divided into Type IV A as a common bile duct cyst combined with intrahepatic bile duct dilation and Type IV B as multiple extrahepatic bile duct cysts without intrahepatic bile duct dilation. Type V is Isolated intrahepatic cystic disease (Caroli disease).^5^ Our case is consistent with a Type IV A choledochal cyst.

The clinical presentation in an adult is non‐specific. The classic triad of choledochal cysts is rarely present in adults, which is supported by our case, where our patient presented only with pain and jaundice but no abdominal mass.^1^ Several imaging methods, including USG, MRI (MRCP), and CT, determine whether a choledochal cyst is present. Ultrasonography is considered the most suitable initial method for evaluating the entire intrahepatic and extrahepatic biliary system, along with the gallbladder. However, it may not always definitively identify the cyst's origin within the bile duct.^1^ While MRCP (Magnetic Resonance Cholangiopancreatography) is considered the gold standard for diagnosing biliary cysts with a high sensitivity ranging from 90% to 100%,^6^, ^7^ in our particular case, we successfully diagnosed our patient using a combination of ultrasonography (USG) and CT Cholangiogram.

Choledochal cysts frequently lead to complications such as cholecystitis, biliary stricture, cholangitis, recurrent acute pancreatitis, cholelithiasis, choledocholithiasis, and even malignancy.^4^ As evidenced by our example, choledocholithiasis is among the most common complications. Adult patients with choledochal cysts should be carefully assessed since biliary malignancy is more common in adults than children. After resection, cancer may develop in the residual stump, but the cyst's excision significantly reduces the risk.^4^

Appropriate management consists of definitive surgery. A study done by Richa et al. in 35 patients with Type IV‐A choledochal cysts found that excision of the extrahepatic part of the cyst and drainage of the intrahepatic half via a wide hilar or sub‐hilar anastomosis produced excellent results.^8^ However, in cases of symptomatic intrahepatic affections (with cholangitis or biliary cirrhosis), treatment should correspond to that used for Type V cysts, with hepatic resection for localized disease and transplantation for diffuse forms.^9^ Saluja et al. discovered that cyst removal with a biliary repair is required to prevent complication recurrence.^10^ Although both hepaticoduodenostomy and Roux‐en‐Y hepaticojejunostomy repair have been documented in the literature, Roux‐en‐Y hepaticojejunostomy is preferred.^11^ The recommended treatment option is complete cyst resection with cholecystectomy and biliary reconstruction with Roux‐en‐Y hepaticojejunostomy or hepaticoduodenostomy.^6^ This surgery succeeded in our patient with no postoperative complications.

## 4 CONCLUSION

Timely diagnosis is crucial in optimizing the prognosis of patients with choledochal cysts, as these conditions are rare and severe and may not always present with the classic triad of symptoms. Comprehensive imaging techniques such as CT, MRCP, or ultrasonography are recommended for early detection. If a choledochal cyst is discovered, the patient should have it surgically removed as soon as possible to avoid complications like choledocholithiasis and malignancy.

Healthcare providers should maintain high vigilance and promptly pursue diagnostic imaging and surgical management when dealing with suspected or confirmed cases of choledochal cysts.

## AUTHOR CONTRIBUTIONS

**Pratik Bhattarai:** Conceptualization; data curation; validation; writing – original draft; writing – review and editing. **Bishowdeep Timilsina:** Conceptualization; data curation; validation; writing – original draft; writing – review and editing. **Prasun Khanal:** Validation; writing – original draft; writing – review and editing.

## FUNDING INFORMATION

No source of funding.

## CONFLICT OF INTEREST STATEMENT

There is no conflict of interest to be declared.

## GUARANTOR

Pratik Bhattarai.

## CONSENT

Written informed consent was obtained from the patient for publication of this case report and accompanying images. A copy of the written consent is available for review by the Editor‐in‐Chief of this journal on request.

## PATIENT PERSPECTIVE

“I am satisfied with the diagnosis and management of my condition.”

## Data Availability

All data underlying the results are available as part of the article, and no additional source data are required.

## References

[1] Anand U , Priyadarshi RN , Kumar B . A giant type IVA choledochal cyst. Ann Gastroenterol. 2012;25(1):73‐75. 24714178 PMC3959351

[2] de Albuquerque V , De Macedo FP , Costa KG , et al. Choledochal cyst‐unusual presentation in the adult phase: case report. Int J Surg Case Rep. 2020;70:33‐36. 32387825 10.1016/j.ijscr.2020.03.014 PMC7210471

[3] Subramony R , Kittisarapong N , Barata I , Nelson M . Choledochal cyst mimicking gallbladder with stones in a six‐year‐old with right‐sided abdominal pain. West J Emerg Med. 2015;16(4):568‐571. 26265970 10.5811/westjem.2015.4.25407 PMC4530916

[4] Kim JY , Kim HJ , Han HY . A case report of an unusual type of choledochal cyst with choledocholithiasis: saccular dilatation of the confluent portion of both intrahepatic ducts. J Korean Soc Radiol. 2015;73(4):252.

[5] Çağan Appak Y , Günşar C , Doğan G , Tarhan S , Kasırga E . The association of choledochal cyst and pancreatitis: a case report and review of the literature. J Pediatr Res. 2017;4(2):78‐81.

[6] Ali A , Gomes RR . A case of choledochal cyst complicated by acute pancreatitis with choledocholithiasis. J Gastroenterol Hepatol Res. 2021;2(1). doi:10.31546/2732-5652.1003

[7] Metcalfe MS , Wemyss‐Holden SA , Maddern GJ . Management dilemmas with choledochal cysts. Arch Surg. 2003;138(3):333‐339. 12611583 10.1001/archsurg.138.3.333

[8] Lal R , Agarwal S , Shivhare R , et al. Type IV‐A choledochal cysts: a challenge. J Hepatobiliary Pancreat Surg. 2005;12(2):129‐134. 15868076 10.1007/s00534-004-0960-1

[9] Cerwenka H . Bile duct cyst in adults: interventional treatment, resection, or transplantation? World J Gastroenterol. 2013;19(32):5207‐5211. 23983423 10.3748/wjg.v19.i32.5207 PMC3752554

[10] Saluja SS et al. Management of choledochal cysts and their complications. Am Surg. 2012;78(3):284‐290. 22524764

[11] Soares KC , Arnaoutakis DJ , Kamel I , et al. Choledochal cysts: presentation, clinical differentiation, and management. J Am Coll Surg. 2014;219(6):1167‐1180. 25442379 10.1016/j.jamcollsurg.2014.04.023 PMC4332770

